# Supplementary material for: Global hypo-methylation in a proportion of glioblastoma enriched for an astrocytic signature is associated with increased invasion and altered immune landscape
Source: eLife. 2022 Nov 22;11:e77335. doi: 10.7554/eLife.77335 (PMC9681209; doi:10.7554/eLife.77335)
Supplement: Figure 2—source data 1. [file elife-77335-fig2-data1.zip › Figure_2_source_data_1/Figure_2A/homerResults/motif10.similar.html]

motif10

## Information for motif10

C
T
G
A
C
A
T
G
A
C
T
G
A
T
C
G
C
G
T
A
C
T
A
G
A
G
T
C
G
C
T
A
A
T
C
G
G
A
C
T
A
C
G
T
C
A
T
G
  
Reverse Opposite:  

G
A
T
C
T
G
C
A
C
T
G
A
T
A
G
C
C
G
A
T
A
C
T
G
A
G
T
C
A
C
G
T
A
T
G
C
G
T
A
C
G
T
A
C
A
G
C
T
  

|  |  |
| --- | --- |
| p-value: | 1e-24 |
| log p-value: | -5.560e+01 |
| Information Content per bp: | 1.755 |
| Number of Target Sequences with motif | 21.0 |
| Percentage of Target Sequences with motif | 0.90% |
| Number of Background Sequences with motif | 1.1 |
| Percentage of Background Sequences with motif | 0.03% |
| Average Position of motif in Targets | 102.7 +/- 57.4bp |
| Average Position of motif in Background | 104.0 +/- 0.0bp |
| Strand Bias (log2 ratio + to - strand density) | 1.0 |
| Multiplicity (# of sites on avg that occur together) | 1.00 |
| Motif File: | file (matrix) reverse opposite |

### Similar de novo motifs found

|  |  |  |  |  |  |  |  |
| --- | --- | --- | --- | --- | --- | --- | --- |
| Rank | Match Score | Redundant Motif | P-value | log P-value | % of Targets | % of Background | Motif file |
| 1 | 0.633 | G T C A G C A T A C T G C T A G A T C G C G T A A C T G A T G C A G C T C G T A | 1e-18 | -41.647817 | 0.90% | 0.07% | motif file (matrix) |
| 2 | 0.659 | G A T C C T A G A C T G A T G C G A C T A T G C G A T C C G A T A G C T T G C A | 1e-16 | -38.831499 | 0.86% | 0.08% | motif file (matrix) |
